# Efficacy and safety analysis of dexamethasone-lipiodol emulsion in prevention of post-embolization syndrome after TACE: a retrospective analysis

**DOI:** 10.1186/s12876-021-01839-w

**Published:** 2021-06-11

**Authors:** Haohao Lu, Chuansheng Zheng, Bin Liang, Bin Xiong

**Affiliations:** 1 Department of Radiology, Wuhan Union Hospital, Tongji Medical College, Huazhong University of Science and Technology, Jiefang Avenue #1277, Wuhan, 430022 China; 2 Hubei Province Key Laboratory of Molecular Imaging, Wuhan, 430022 China

**Keywords:** Dexamethasone, Lipiodol emulsion, Post-embolization syndrome, TACE, Transarterial chemoembolization, Hepatocellular carcinoma

## Abstract

**Background:**

To investigate the efficacy and safety of dexamethasone-lipiodol emulsion in the prevention of post-embolization syndrome after TACE.

**Method:**

The data of 255 patients who underwent TACE in the interventional department from June 2017 to June 2020 were collected. This is a retrospective assessment of patients who were non-randomly treated with dexamethasone in TACE. The patients were divided into two groups: TACE using lipiodol + chemotherapeutic emulsion group (TACE group, N = 133); TACE using lipiodol + dexamethasone + chemotherapeutic emulsion group (TACE + dexamethasone group, N = 122). Primary study endpoint: incidence of abdominal pain, fever, nausea and vomiting 0–72 h after TACE in both groups. Secondary study endpoints: incidence of infection after TACE in both groups.

**Results:**

Incidence of post-embolization syndrome after TACE (TACE group vs TACE + dexamethasone group): abdominal pain, 55.6% versus 36.1% (*P* value 0.002); fever, 37.6% versus 13.1% (*P* value 0.000); nausea, 60.9% versus 41.0% (*P* value 0.001); vomiting, 48.1% versus 21.3% (*P* value 0.000). Incidence of infection after TACE (TACE group vs TACE + dexamethasone group): 1.5% versus 2.5% (*P* value 0.583).

**Conclusion:**

The lipiodol + dexamethasone emulsion can significantly reduce the incidence rate of post-embolization syndrome after TACE, with exact effect and high safety.

## Introduction

Primary hepatocellular carcinoma is one of the malignant tumors with high morbidity and mortality worldwide [1–3], which is a serious threat to people's life and health. Because there are no obvious specific symptoms and signs in the early stage of the disease, most patients are found in the middle and advanced stages and lose the chance of surgery. Transcatheter arterial chemoembolization (TACE), It has been performed since the late 1970s [4, 5]. TACE is currently one of the effective means of treating advanced hepatocellular carcinoma, which significantly prolongs the OS and PFS of patients and benefits an increasing number of patients with hepatocellular carcinoma [6, 7]. The most common side effects after TACE are post-embolization syndrome [8, 9], including abdominal pain, fever, nausea and vomiting. Post-embolization syndrome will increase the suffering of patients, aggravate the physical and psychological burden of patients and reduce the compliance of patients with treatment. At the same time, it prolongs the patient’s hospital stay and increases the patient's medical costs [10]. Because patients with hepatocellular carcinoma are mostly complicated with liver cirrhosis, many patients are complicated with gastric esophageal varices, severe post-embolization syndrome, especially nausea and vomiting, which may lead to gastrointestinal bleeding caused by gastric esophageal varices rupture, and even lead to death of patients. Therefore, prevention of post-embolic syndrome appears crucial. The aim of this study was to investigate the efficacy and safety of dexamethasone-lipiodol emulsion in the prevention of post-embolization syndrome after TACE.

## Materials and methods

### General information

The data of 255 patients who underwent TACE in the Department of Intervention, Wuhan Union Hospital, Tongji Medical College, Huazhong University of Science and Technology from June 2017 to June 2020 were collected. Inclusion criteria: (1) clinical or pathological diagnosis of primary hepatocellular carcinoma; (2) liver function classification: Child–Pugh classification A or B, performance score (ECOG) 0–1 points; (3) no use of molecular targeted drugs or immunotherapy during treatment; (4) age range: 18–70 years. Exclusion criteria: (1) liver function classification: Child–Pugh classification C, performance score (ECOG) ≥ 2 points; (2) severe coagulation dysfunction and can not be corrected; (3) cachexia or extensive distant metastasis of the tumor; (4) complete occlusion of the portal vein and few collateral vessels; (5) renal insufficiency. This is a retrospective assessment of patients who were non-randomly treated with dexamethasone in TACE. There are two medical groups in our interventional therapy department. One of the medical groups routinely used dexamethasone in TACE to prevent the occurrence of post-embolization syndrome. Another medical group did not use dexamethasone in TACE. According to whether dexamethasone has been used in TACE, the patients were divided into two groups: TACE using lipiodol + chemotherapeutic emulsion group (TACE group, N = 133); TACE using lipiodol + dexamethasone + chemotherapeutic emulsion group (TACE + dexamethasone group, N = 122). The collected baseline data before TACE included gender, age, etiology of liver cirrhosis, ALT, AST, ALP, preoperative Child–Pugh classification of liver function, BCLC staging of tumor, whether opioid analgesics were used before operation, whether the patient was fasting before operation, previous history of vomiting, history of motion sickness, history of smoking, history of drinking, types of chemotherapeutic drugs used during TACE, and dosage of lipiodol used during TACE.

### Method

After disinfection, draping, and local anesthesia of the puncture site with 2% lidocaine, the right femoral artery was punctured using the Seldinger technique and a 5F vascular sheath was placed. The feeding artery of the tumor was identified by catheterization with a 5F Yashino catheter to the celiac trunk and superior mesenteric artery for angiography. A 2.7 F microcatheter was then used to superselectively cannulate into the tumor feeding artery. Embolization was performed by slowly injecting an appropriate amount of lipiodol emulsion and supplementing embolization with 300–500 um gelatin sponge particles, and the embolization endpoint was stagnation of forward blood flow in the tumor feeding artery. Chemotherapeutic drugs used during surgery are divided into two types: (1) lobaplatin 50 mg; (2) epirubicin 30 mg. The amount of iodized oil used was 5–20 ml. Composition of lipiodol emulsion in TACE group: lipiodol + chemotherapeutic drugs; Composition of lipiodol emulsion in TACE + dexamethasone group: lipiodol + dexamethasone 10 mg + chemotherapeutic drugs.

Materials and drugs used for TACE: 5F vascular sheath (TERUMO5F-10CM, Terumo, Japan), 0.035 inch (RFGA35153M, Terumo, Japan), 5F Yashino catheter (Terumo, Japan), 2.7F microcatheter (Terumo, Japan). Lobaplatin (GYZZ H20050308, Hainan Chang’an International Pharmaceutical Co., Ltd.), epirubicin (GYZZ H19990280, Zhejiang Hisun Pharmaceutical Co., Ltd.), Dexamethasone Sodium Phosphate for Injection (GYZZ H20080355, Chongqing Laimei Pharmaceutical Co., Ltd.).

### Outcome measures

*Primary study endpoint* incidence of abdominal pain, fever, nausea and vomiting 0–72 h after TACE in both groups.

*Secondary study endpoints* incidence of infection after TACE in both groups.

Fever was defined as an axillary temperature > 37.3 °C. Nausea was defined as an uncomfortable feeling of wanting to vomit, but without contractile movements of the abdominal and diaphragmatic muscles. Vomiting is defined as contraction of the diaphragm, pectoral, and abdominal muscles, which may or may not be accompanied by vomiting of gastric contents. The evaluation criteria for nausea and vomiting were used: Common Terminology Criteria for Adverse Events (CTCAE 5.0) [11]. Infection includes positive blood cultures or liver abscess.

### Statistical methods

Statistical analysis was performed using SPSS software (Version 24.0, IBM, Armonk, New York). Number of cases (expressed as percentage) was used for enumeration data, and chi-square test was used for differences, including Pearson Chi-Square and Fisher’s Exact Test. Measurement data were expressed as mean ± standard deviation, and two independent samples *t* test was used. *P* < 0.05 was considered to indicate a statistically significant difference.

## Results

### Basic information

Preoperative enumeration data of TACE group and TACE + dexamethasone group: gender, preoperative Child–Pugh classification of liver function, etiology of liver cirrhosis, BCLC stage of tumor, previous history of vomiting, history of motion sickness, smoking history, drinking history, preoperative use of opioid analgesics, preoperative fasting (Table 1). Chi-square test was used between two groups with *P* value > 0.05 and no statistical difference.

**Table 1:** General information of the patients

	Group		Chi-square test (*P* value)	
	TACE (N = 133)	TACE + Dexamethasone (N = 122)	Pearson chi-square	Fisher’s exact test
Gender				
Female				
Count (%)	68 (51.1%)	52 (42.6%)	0.174	0.209
Male				
Count (%)	65 (48.9%)	70 (57.4%)		
Child–Pugh classification of liver function				
A				
Count (%)	95 (71.4%)	85 (69.7%)	0.758	0.784
B				
Count (%)	38 (28.6%)	37 (30.3%)		
Etiology of cirrhosis				
Hepatitis B				
Count (%)	120 (90.2%)	111 (91.0%)	0.836	1.000
Hepatitis C				
Count (%)	13 (9.8%)	11 (9.0%)		
BCLC staging				
A				
Count (%)	14 (10.5%)	12 (9.8%)	0.682	
B				
Count (%)	80 (60.2%)	68 (55.7%)		
C				
Count (%)	39 (29.3%)	42 (34.4%)		
History of vomiting				
No				
Count (%)	108 (81.2%)	95 (77.9%)	0.509	0.537
Yes				
Count (%)	25 (18.8%)	27 (22.1%)		
Motion sickness				
No				
Count (%)	100 (75.2%)	99 (81.1%)	0.251	0.290
Yes				
Count (%)	33 (24.8%)	23 (18.9%)		
Smoking history				
No				
Count (%)	105 (78.9%)	100 (82.0%)	0.544	0.636
Yes				
Count (%)	28 (21.1%)	22 (18.0%)		
Alcohol history				
No				
Count (%)	110 (82.7%)	103 (84.4%)	0.712	0.738
Yes				
Count (%)	23 (17.3%)	19 (15.6%)		
History of analgesics				
No				
Count (%)	93 (69.9%)	90 (73.8%)	0.496	0.578
Yes				
Count (%)	40 (30.1%)	32 (26.2%)		
Preoperative fasting				
No				
Count (%)	65 (48.9%)	69 (56.6%)	0.220	0.259
Yes				
Count (%)	68 (51.1%)	53 (43.4%)		

Preoperative measurement data of TACE group and TACE + dexamethasone group: age, ALT, AST and ALP (Table 2). Comparisons between two groups were performed using the *t* test with a *P* value > 0.05 and no statistical difference.

**Table 2:** Patient’s age and liver function before TACE

Group	Mean	Std. deviation	*t* test (*P* value)
Age			
TACE	47.3	11.6	0.331
TACE + dexamethasone	48.8	12.5	
ALT			
TACE	42.6	17.8	0.155
TACE + dexamethasone	47.1	19.0	
AST			
TACE	41.3	17.8	0.093
TACE + dexamethasone	45.4	17.7	
ALP			
TACE	90.7	29.1	0.389
TACE + dexamethasone	93.6	23.7	

Intraoperative enumeration data of TACE group and TACE + dexamethasone group: types of chemotherapeutic drugs used in TACE and dosage of lipiodol used in TACE (Table 3). Chi-square test was used for comparison between two groups with *P* value > 0.05 and no statistical difference.

**Table 3:** Application of chemotherapeutic drugs and lipiodol in TACE

	Group		Chi-square test (*P* value)	
	TACE (N = 133)	TACE + dexamethasone (N = 122)	Pearson chi-square	Fisher’s exact test
Intraoperative chemotherapeutic agents				
Lobaplatin				
Count (%)	68 (51.1%)	63 (51.6%)	0.935	1.000
Epirubicin				
Count (%)	65 (48.9%)	59 (48.4%)		
Intraoperative lipiodol dose				
< 5 ml				
Count (%)	41 (30.8%)	39 (32.0%)	0.897	
5–10 ml				
Count (%)	55 (41.4%)	47 (38.5%)		
10 ml				
Count (%)	37 (27.8%)	36 (29.5%)		

### Incidence of post-embolization syndrome after TACE (Table 4)

**Table 4:** Incidence of Post embolism syndrome after TACE

	Group		Chi-square test (*P* value)	
	TACE (N = 133)	TACE + Dexamethasone (N = 122)	Pearson Chi-Square	Fisher's exact test
Abdominal pain				
No				
Count (%)	59 (44.4%)	78 (63.9%)	0.002	0.002
Yes				
Count (%)	74 (55.6%)	44 (36.1%)		
Fever				
No				
Count (%)	83 (62.4%)	106 (86.9%)	0.000	0.000
Yes				
Count (%)	50 (37.6%)	16 (13.1%)		
Nausea				
No				
Count (%)	52 (39.1%)	72 (59.0%)	0.001	0.002
Yes				
Count (%)	81 (60.9%)	50 (41.0%)		
Vomiting				
No				
Count (%)	69 (51.9%)	96 (78.7%)	0.000	0.000
Yes				
Count (%)	64 (48.1%)	26 (21.3%)		

Incidence of post-embolization syndrome after TACE (TACE group vs TACE + dexamethasone group): abdominal pain, 74 (55.6%) versus 44 (36.1%), *P* value 0.002; fever, 50 (37.6%) versus 16 (13.1%), *P* value 0.000; nausea, 81 (60.9%) versus 50 (41.0%), *P* value 0.001/0.002; vomiting, 64 (48.1%) versus 26 (21.3%), *P* value 0.000.

Chi-square test was used for comparison between the two groups, including Pearson Chi-Square and Fisher’s Exact Test, with *P* value < 0.05, which was statistically different.

### Incidence of infection after TACE (Table 5)

**Table 5:** Incidence of infection after TACE

	Group		Chi-square test (*P* value)	
	TACE (N = 133)	TACE + Dexamethasone (N = 122)	Pearson chi-square	Fisher’s exact test
Infection				
No				
Count (%)	131 (98.5%)	119 (97.5%)	0.583	0.672
Yes				
Count (%)	2 (1.5%)	3 (2.5%)		

Incidence of infection after TACE (TACE group vs TACE + dexamethasone group): 2 (1.5%) versus 3 (2.5%), *P* value 0.583/0.672.

Chi-square test was used for comparison between the two groups, including Pearson Chi-Square and Fisher’s Exact Test, with *P* value > 0.05 and no statistical difference.

## Discussion

TACE is one of the main means of treating hepatocellular carcinoma [37]. TACE was shown to improve median survival from 16 to 20 months. TACE is effective in the treatment of hepatocellular carcinoma and plays a very important role in the treatment of hepatocellular carcinoma. The most common side effects after TACE are post-embolization syndrome, including abdominal pain, fever, nausea and vomiting. More and more attention has been paid to post-embolization syndrome after TACE by doctors and patients. According to the existing studies on the efficacy and safety of TACE, the incidence rate of post-embolization syndrome after TACE is about 47.7% [6].

The causes of post-embolization syndrome after TACE are [12–14]: (1) the trauma, ischemia, and hypoxia of the operation lead to pain; the aseptic inflammation caused by ischemia and hypoxia induces the release of a variety of transmitters, leading to fever, nausea and vomiting; (2) the intraoperative use of chemotherapeutic drugs, through the blood circulation, stimulates the chemoreceptors of the gastrointestinal tract; in particular, chemotherapeutic drugs are often injected through the celiac trunk or superior mesenteric artery branches during TACE, while the celiac trunk or superior mesenteric artery already has vascular branches that directly supply the gastrointestinal tract; the epirubicin and platinum used during TACE are chemotherapeutic drugs with moderate to high emetic risk; (3) the organs involved in TACE are the liver and gallbladder, and hepatic arterial embolization will lead to hepatobiliary ischemia; Similar to the causes of PONV, hepatobiliary surgery is more likely to cause postoperative nausea and vomiting than other surgeries; (4) the stress during TACE may cause the release of dopamine, epinephrine and other transmitters, resulting in nausea and vomiting; (5) patient factors, such as young women, no smoking history, history of PONV, history of motion sickness; (6) tumor ischemic necrosis after TACE, metabolism and absorption of necrotic substances, and release of inflammatory transmitters, resulting in pain and fever. Severe post-embolization syndrome will increase the suffering of patients, aggravate the physical and psychological burden of patients, and reduce the compliance of patients with treatment. At the same time, it prolongs the patient's hospital stay and increases the patient’s medical costs [6, 15].

Post-embolization syndrome after TACE is mainly controlled by symptomatic treatment. Studies have confirmed that 5-HT3 receptor antagonists can effectively control the occurrence of nausea and vomiting after TACE. The application of analgesic drugs after TACE can reduce the occurrence of postoperative abdominal pain in patients. However, because most patients with primary hepatocellular carcinoma have a background of cirrhosis, they may have liver dysfunction before TACE. After TACE, the use of embolic agents and chemotherapeutic drugs may cause short-term liver function damage. At this time, if multiple drugs are used concomitantly to control post-embolization syndrome, the burden of liver metabolism will be increased, resulting in aggravated liver function damage. It is well-known that dexamethasone, as a steroid preparation, has anti-infective and immunosuppressive effects. Dexamethasone, like other glucocorticoids, has pharmacological effects such as inhibition of immunity, anti-shock and enhancement of stress response, so it is widely used in the treatment of a variety of diseases. It has (1) anti-inflammatory effect: it can reduce and prevent the tissue response to inflammation, thereby reducing the performance of inflammation. Hormones inhibit the accumulation of inflammatory cells, including macrophages and leukocytes, at sites of inflammation, and inhibit phagocytosis, release of lysosomal enzymes, and synthesis and release of inflammatory chemical mediators. (2) Immunosuppressive effects: including preventing or inhibiting cell-mediated immune responses, delayed allergic reactions, reducing the number of T lymphocytes, monocytes, and eosinophils, reducing the binding ability of immunoglobulins to cell surface receptors, and inhibiting the synthesis and release of interleukins, thereby reducing the transformation of T lymphocytes into lymphoblasts, and reducing the expansion of the primary immune response. It can reduce the passage of immune complexes through the basement membrane, and can reduce the concentration of complement components and immunoglobulin. Dexamethasone has been shown to reduce the incidence of side effects induced by emetic chemotherapy. Dexamethasone is targeted at glucocorticoid receptors, which play a very important role in inflammation and immune response by inducing apoptosis of immune cells and inhibiting the release of inflammatory transmitters. Studies have shown that dexamethasone has a membrane-stabilizing effect, which can not only maintain the integrity of lysosomal membranes, but also regulate vascular permeability by strengthening cell–cell contact [16]. Dexamethasone plays a very important role in regulating systemic and local inflammatory responses due to its powerful role in stabilizing the endothelium. Many studies have shown [17] that dexamethasone plays a very effective role in preventing PONV and CINV. Liu et al. [18] reported that dexamethasone could significantly reduce the incidence of POV in postoperative and few adverse effects were reported. Bustos et al. [19] found that dexamethasone is a safe adjunct to perioperative protocol that may reduce nausea, thus improving patient satisfaction, and there is an associated reduction in length of stay that may reduce cost of hospitalization. The findings of Moheimani et al. [20] showed that a single preoperative dose of i.v. dexamethasone reduces PONV. Dexamethasone also plays a very important role in the prevention and treatment of CINV [21]. Isoda et al. [22] found that the three-antiemetic regimen consisting of palonosetron, aprepitant, and dexamethasone was safe and effective for controlling CINV. The study of Lorusso et al. [23] showed that a single dose of palonosetron and dexamethasone was able to prevent CINV in most patients. Celio et al. [24] demonstrated that palonosetron plus 1-day dexamethasone regimen provides a valid treatment option for prevention of CINV. Studies have shown that dexamethasone relieves postoperative pain after surgery and also prolongs the duration of postoperative analgesia [25, 26]. The article by Tenghui Zhang et al. [27] reported that a single, intravenous 8-mg dose of dexamethasone upon induction of anesthesia reduced the intensity of postoperative pain, and shortened the postoperative length of stay. Klag et al. [28] reported that dexamethasone decreases opioid requirements in the first 24 h following surgery, provides improved pain control, and decreases antiemetic use, Dexamethasone is an important multimodal adjunct for controlling pain and postoperative nausea and vomiting. Perioperative dexamethasone is effective to reduce the pain, nausea and vomiting after thyroid surgery, which was reported by Cheng et al. [29]. Some studies have reported [30] not only does Dexamethasone reduce the incidence of PONV but also decreases postoperative pain. Because of this, we speculated that dexamethasone may also have very good safety and efficacy in controlling post-embolization syndrome after TACE.

In recent years, some clinical trials have also confirmed the utility and safety of dexamethasone in preventing the adverse effects of TACE [31–33]. Ogasawara et al. [34] found that the dexamethasone regimen was more effective than the control regimen at preventing TACE-induced fever, anorexia, and nausea/vomiting in patients with HCC. Intra-arterial lidocaine, steroids, and a 5-HT3 receptor antagonist are found to offer potential benefit in the management of PES symptoms by Blackburn et al. [35]. Ogasawara et al. [34] showed that dexamethasone could reduce the incidence of post-embolization syndrome after TACE and promote the recovery of patients. Meanwhile, dexamethasone was also very safe in patients with diabetes and impaired glucose tolerance. In the study, it was found that for patients with hepatitis B background, the use of dexamethasone also did not lead to the activation of hepatitis B. The results of this study showed that: Incidence of post-embolization syndrome after TACE (TACE group vs TACE + dexamethasone group): abdominal pain, 55.6% versus 36.1% (*P* < 0.05); fever, 37.6% versus 13.1% (*P* < 0.05); nausea, 60.9% versus 41.0% (*P* < 0.05); vomiting, 48.1% versus 21.3% (*P* < 0.05). All were statistically different. The incidence of post-embolization syndrome in TACE + dexamethasone group was significantly lower than that in TACE group.

Since dexamethasone can inhibit the immune function of the body and has no antibacterial effect, its clinical use can induce infection or aggravate infection, which can make the potential foci of infection in the body spread or the quiescent foci of infection resurface, especially those with decreased original resistance. Since patients often feel good about themselves after using dexamethasone and mask the symptoms of infection development, the use of dexamethasone in TACE has the potential to increase the risk of infection in patients. However, existing studies [36] showed that the use of dexamethasone in the treatment of post-embolization syndrome after TACE did not increase the incidence of infection in patients, and the use of dexamethasone in TACE was safe. In this study, the incidence of infection after TACE: 2 cases in TACE group, with an incidence rate of 1.5%. 3 cases in the TACE + dexamethasone group, with an incidence rate of 2.5%. (*P* > 0.05) There was no significant statistical difference, which was consistent with the results of other studies. However, in other studies, the administration mode of dexamethasone was intravenous bolus injection. In this study, the administration mode of dexamethasone was emulsion mixed with lipiodol. The microcatheter was used for injection into the tumor area, which was also safe and effective.

## Conclusions

There are multiple causes for post-embolization syndrome after TACE, including surgical trauma, ischemia and hypoxia of tumor tissue, aseptic inflammation, intraoperative use of chemotherapeutic drugs, ischemia of liver and bile duct, stress during TACE, patient factors (such as young women, no smoking history, history of PONV, history of motion sickness), absorption and metabolism of necrotic substances after tumor embolization. In TACE, the lipiodol + dexamethasone emulsion can significantly reduce the incidence rate of post-embolization syndrome after TACE, with exact effect and high safety. The preventive intervention in TACE can reduce the psychological and physical burden of patients, reduce the perioperative risk and improve the treatment compliance of patients. The shortcomings of this study are that the data are from a single-center retrospective study, and the sample size is limited. The practice differences of the two medical groups may affect the observed results. A multi-center, large-sample, prospective study is feasible at a later stage to provide more help for clinical work. At the same time, in this study, the use of dexamethasone was to inject lipiodol + dexamethasone emulsion during embolization, and relevant studies could be designed at a later stage to investigate the efficacy analysis of lipiodol + dexamethasone emulsion embolization and conventional intravenous bolus injection of dexamethasone.

## Data Availability

The datasets used and analysed during the current study are available from the corresponding author on reasonable request.
